# Geographic disparities impacting oral vaccine performance: Observations and future directions

**DOI:** 10.1093/cei/uxae124

**Published:** 2025-01-21

**Authors:** Rachel M. Burke, Sasirekha Ramani, Julia Lynch, Laura V. Cooper, Haeun Cho, Ananda S. Bandyopadhyay, Carl D. Kirkwood, A. Duncan Steele, Gagandeep Kang

**Affiliations:** 1. Bill & Melinda Gates Foundation, Seattle, WA, USA; 2. Baylor College of Medicine, Houston, TX, USA; 3. International Vaccine Institute, Seoul, Republic of Korea; 4. Imperial College London, London, UK

**Keywords:** Vaccination, Vaccine, Antibodies

## Abstract

Oral vaccines have several advantages compared with parenteral administration: they can be relatively cheap to produce in high quantities, easier to administer, and induce intestinal mucosal immunity that can protect against infection. These characteristics have led to successful use of oral vaccines against rotavirus, polio, and cholera. Unfortunately, oral vaccines for all three diseases have demonstrated lower performance in the highest-burden settings where they are most needed. Rotavirus vaccines are estimated to have >85% effectiveness against hospitalization in children <12 months in countries with low child mortality, but only ~65% effectiveness in countries with high child mortality. Similarly, oral polio vaccines have lower immunogenicity in developing country settings compared with high-resource settings. Data are more limited for oral cholera vaccines, but suggest lower titers among children compared with adults, and, for some vaccines, lower efficacy in endemic settings compared with non-endemic settings. These disparities are likely multifactorial, and available evidence suggests a role for maternal factors (e.g., transplacental antibodies, breastmilk), host factors (e.g., genetic polymorphisms—with the best evidence for rotavirus—or previous infection), and environmental factors (e.g., gut microbiome, coinfections). Overall, these data highlight the rather ambiguous and often contradictory nature of evidence on factors affecting oral vaccine response, cautioning against broad extrapolation of outcomes based on one population or one vaccine type. Meaningful impact on performance of oral vaccines will likely only be possible with a suite of interventions, given the complex and multifactorial nature of the problem and the degree to which contributing factors are intertwined.

## SUMMARY

Oral vaccines have been developed and used with much success globally. However, despite their undeniable impact, oral vaccines against rotavirus, polio, and cholera do not always perform equally across different populations. In this article, we summarize the state of oral vaccines for these diseases and discuss potential hypotheses for performance disparities, including maternal and infant factors, environmental factors, and vaccine characteristics.

## Graphical Abstract

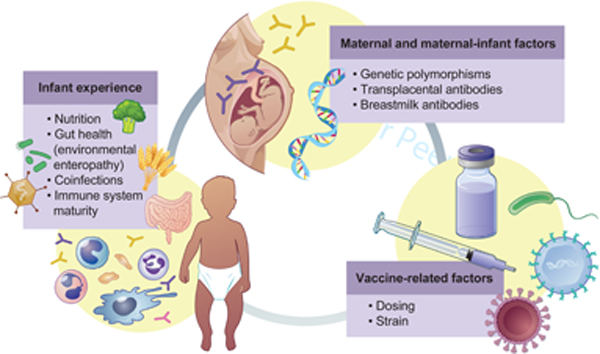

## Introduction

Orally administered vaccines have been in use since the late 1950s, when the live, attenuated Sabin oral polio vaccine (OPV) was first introduced for mass vaccination campaigns [1]. Since then, oral vaccines have been developed for other diseases transmitted via the fecal-oral route, most notably rotavirus and cholera [2]. Oral vaccines have several advantages compared with parenteral administration: they can be cheaper to produce in high quantities (e.g., OPV is <1/5 the cost of inactivated polio vaccine), easier to administer, and have the ability to induce intestinal mucosal immunity that can protect against infection [2, 3]. Indeed, the drastic reduction in poliomyelitis cases and poliovirus circulation globally since the 1950s is largely attributed to widespread use of OPV in routine immunization programs and outbreak response activities [4]. Rotavirus vaccines have also demonstrated success in reducing global burden of disease, and introduction of rotavirus vaccination into routine immunization programs is estimated to have prevented 139,000 under-5 deaths during 2006 – 2019, before introduction in large countries with high burden such as India, Pakistan, Nigeria, and DRC [5]. In contrast to rotavirus and polio vaccines, which are given in many countries as standard preventive care for infants and young children, oral cholera vaccines (OCVs) are given only in campaign modality (i.e., via mass administration during discrete periods in high-risk areas). OCVs are used primarily in outbreak settings and in endemic countries, and have the ability to induce herd protection, stopping outbreaks and averting cases [6, 7]. Despite these successes, oral vaccines for all three diseases have demonstrated lower performance in the highest-burden settings where they are most needed. In the present review, we will introduce these three pathogens and their vaccines, summarize vaccine performance disparities, and present evidence for contributing factors.

## Pathogen Overview

### Rotavirus

Rotavirus is a leading cause of severe acute gastroenteritis in children under the age of five years. Prior to the introduction of live-attenuated oral rotavirus vaccines, nearly every child experienced a rotavirus infection by the age of 5 years, leading to over 2 million hospitalizations and an estimated 528,000 deaths each year [8, 9]. Vaccine introduction in over 120 countries since 2006 has resulted in substantial reductions in the burden of rotavirus-associated morbidity and mortality. However, an estimated 108,470 deaths were still attributable to rotavirus in 2021, with nearly all occurring in low and middle-income countries and over 70% occurring in the WHO African region [10].

Rotaviruses are non-enveloped, double stranded (ds) RNA viruses within the family *Sedoreoviridae.* The 11 segments of dsRNA code for six structural proteins (viral proteins, VP) and six nonstructural proteins. Traditionally, rotavirus is classified into G- and P-types based on sequence differences in the outer capsid proteins VP7 and VP4, respectively. Recently, a more comprehensive typing system based on the whole genome has been established. The first human rotavirus was visualized in thin section electron micrographs of duodenal biopsies from children with gastroenteritis in 1973 [11]. Shortly thereafter, the virus was identified in stool samples of children with diarrhea [12] and subsequently recognized as the major cause of pediatric gastroenteritis worldwide. The spectrum of clinical presentations is broad, ranging from asymptomatic infections and mild diarrhea to severe, life-threatening gastroenteritis that may require hospitalization. Dehydration, electrolyte imbalance, and death may occur if adequate and timely fluid replacement is not provided. Rotavirus pathogenesis is multifactorial and involves both malabsorptive and secretory mechanisms of diarrhea [13–15]. Rotavirus infection also results in the secretion of serotonin that can alter gut motility and activate vomiting centers in the brain [16]. Rotavirus infection and disease are not limited to the gastrointestinal tract, and extraintestinal complications involving the central nervous system can occur [17].

### Poliovirus

In the decades before polio vaccine introduction, polioviruses caused hundreds of thousands of children to become paralyzed each year. Due to a coordinated global effort to vaccinate children, cases of paralysis have dropped by over 99%, and two out of three wild poliovirus (WPV) serotypes have been eradicated [1]. Despite this tremendous progress, endemic WPV1 circulation continues in Afghanistan and Pakistan in 2024, while other geographies have experienced outbreaks due to reverted vaccine-derived virus [18]. Several factors contribute to the complexity of polio eradication, including viral and vaccine characteristics in addition to conflict settings and cultural factors that complicate vaccination efforts in certain geographies.

Polioviruses are non-enveloped, single-stranded RNA viruses of the *Picornaviridae* family, existing in three serotypes (1, 2, 3). The poliovirus mRNA is translated as one polypeptide which undergoes protease cleavage into 10 viral proteins. Poliovirus transmission occurs primarily through the fecal-oral route in lower-sanitation settings, with the oral-oral route gaining primacy in higher-sanitation settings [19]. Viruses replicate initially in the oropharyngeal and intestinal mucosa and are shed in the feces. In the majority of infections, viral replication is generally confined to the gastrointestinal tract and a transient viremia, and symptoms are either non-existent or nonspecific (e.g., fever, malaise) [20]. However, in rare cases (~1 – 2%), a prolonged secondary viremia can provide the opportunity for the virus to infect the central nervous system via retrograde axonal transport or by directly crossing the blood-brain barrier [20, 21]. Infection of motor neurons can lead to limb paralysis or even death, when breathing muscles are affected. No cure is available, and treatment of paralysis is limited to physical therapy or mechanical ventilation where indicated [19].

### Cholera

In 2015, annual cholera burden was estimated as 1.3 – 4 million cases per year and 21,000 – 143,000 related deaths [22]. With the exception of sporadic cases associated with ingestion of uncooked shellfish in coastal areas in a few high-income countries, cholera infection and disease are almost exclusively associated with extreme poverty, lack of access to clean water, and inadequate sanitation [23]. Climate change together with other factors (e.g., political conflict, forced migration, and economic and social disruptions induced by the COVID-19 pandemic) have further propagated cholera outbreaks since 2020, and as of January 31, 2024, a new surge in infections had been reported in at least 30 countries [24]. Notably, several reporting countries were either non-endemic countries (e.g., Lebanon and Syria) or had not been affected by cholera for a long period of time (e.g., Haiti and Dominican Republic). In January 2023, WHO raised the global cholera crisis to a grade 3 emergency, the highest level of the grading system [25].

Cholera, a diarrheal disease caused by *Vibrio cholerae,* is spread from person to person by the fecal-oral route or through contaminated food or water. In its most severe form, affected individuals have voluminous watery stools which can lead to dehydration and death within hours, even in otherwise healthy adults, if prompt rehydration is not initiated [26]. After an infectious dose (10^5^ – 10^8^ organisms [27]) is ingested, this non-invasive pathogen colonizes the small intestine and causes illness principally through excretion of a cholera toxin, CTX, that leads to a secretory diarrhea [28]. There are two serogroups associated with severe human disease, O1 and O139; 99% of disease globally is attributed to the O1 serogroup, which has two serotypes, Inaba and Ogawa [29]. Although serologically distinguishable, because of a high degree of similarity in their outer surface polysaccharide, the O-specific polysaccharide (OSP), they are highly cross reactive and indistinguishable in disease caused. Both natural history studies and human challenge studies have confirmed they are also cross protective (i.e., infection with either confers a period of homologous and heterologous protection) [30]. Serum vibriocidal antibody has long been recognized as a correlate of protection against cholera, with OSP as the most influential antigen [31].

## Vaccines and Vaccine Performance

### Rotavirus

**Figure 1:**
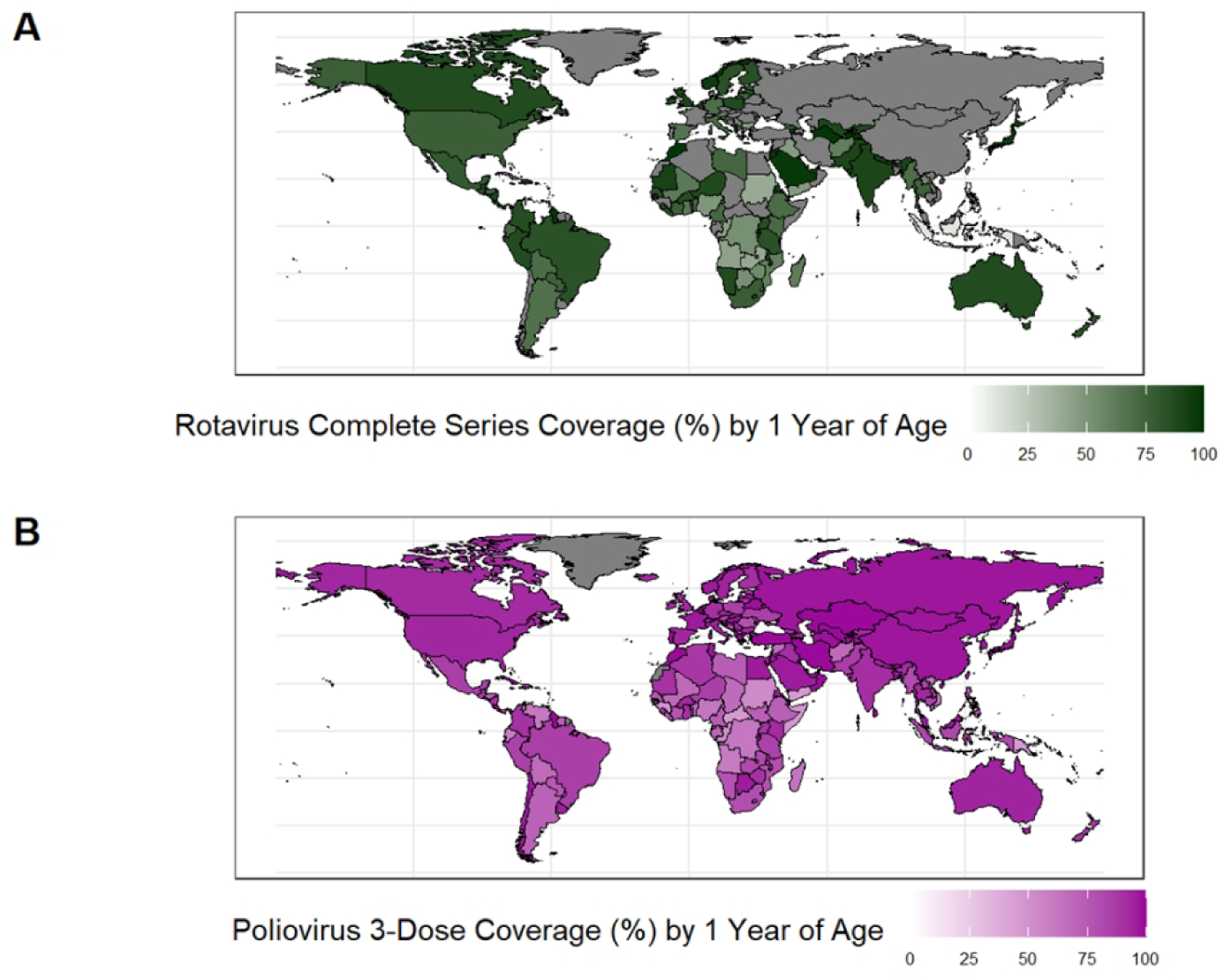
Rotavirus vaccine complete-series coverage (A) and Polio vaccine 3-dose coverage (B) among children by 1 year of age, 2023

Currently, four oral rotavirus vaccines (Rotarix, RotaTeq, Rotavac, and Rotasiil) are prequalified by the World Health Organization (WHO) for global use, meaning that quality, safety, and efficacy data have been reviewed by WHO, and these products have been deemed acceptable for global procurement. Three additional vaccines are nationally licensed in Vietnam and China (Rotavin-M1 in Vietnam and Lanzhou Lamb Rotavirus [LLR] and LLR3 vaccines in China ) (Table 1). All rotavirus vaccines are based on live, attenuated strains; RotaTeq and Rotasiil are reassorted bovine-human viruses, Rotarix and Rotavin are based on human strains attenuated via passage in Vero cells, Rotavac is based on a naturally attenuated bovine-human neonatal strain, LLR is based on a lamb strain, and LLR3 is based on a human-lamb reassortant strain [32, 33]. Several additional vaccines are in preclinical and clinical development, including an oral vaccine to be delivered on a neonatal schedule [33]. Despite inclusion in the national immunization programs of over 120 countries, global rotavirus vaccine coverage in 2023 was only 55%, with averages of 59%, 66% and 58% for high-, lower middle-, and low-income countries respectively (Figure 1) [34].

Rotavirus vaccines are estimated to have averted >139,000 deaths [5] since their introduction in 2006, and >500,000 hospitalizations in 2019 alone [35], among children <5. Since 2019, several countries with large birth cohorts (DRC, Nigeria, India, Indonesia) have introduced rotavirus vaccines into their national schedules [34], suggesting that impact might be even greater if measured in 2024. Despite this tremendous success, there are geographic gaps in vaccine performance. In high-income countries such as the United States and Finland, where initial clinical trials of Rotarix and RotaTeq took place, efficacy of rotavirus vaccines against severe rotavirus gastroenteritis in the first year of life was >90% [36, 37]. However, later clinical trials conducted in sub-Saharan Africa and Asia, including trials of the newer vaccines, Rotasiil and Rotavac, demonstrated markedly lower efficacy of <70% (and even <40%) [38–43].

Performance disparities are also evident in real-world usage. One meta-analysis estimated Rotarix vaccine effectiveness among children <12 months of age to be 86% (95% CI: 81–90%) in countries with low child mortality, 77% (66–85%) in countries with medium child mortality, and 63% (54–70%) in countries with high child mortality; for RotaTeq, vaccine effectiveness was 86% (76–92%) in countries with low child mortality and 66% (51–76%) in countries with high child mortality [44]. Among children in their second year of life, Rotarix vaccine effectiveness was again estimated as lower in countries with high child mortality, compared to countries with low child mortality; sparse data precluded estimates for RotaTeq. Evaluation of vaccine impact (decrease in rotavirus-associated hospitalizations following rotavirus vaccine introduction) also showed differences by child mortality stratum, with the greatest impact in low-mortality settings (78% reduction among children <12 months of age; inter-quartile range [IQR]: 65–85%), and lower impact in higher-mortality settings (55%; 41 – 64%) [45]. Among all children <5 years, impact estimates were somewhat lower, and disparities were somewhat lessened (66% [IQR 49–76%] reduction in the low-mortality setting vs. 50% [IQR, 41–65%] in the highest-mortality setting). Despite the fact that higher-mortality settings may show a lower estimated impact of rotavirus vaccination when measured via percent reduction, their high baseline burden can result in a greater number of hospitalizations and deaths averted when compared with lower-mortality settings [38].

Due to their more recent introduction, effectiveness and impact data are not readily available for Rotavac and Rotasiil. However, given their similarities to existing vaccines, it is expected that performance differences would be similar as with efficacy estimates. Indeed, preliminary effectiveness data from studies in India suggest performance in line with above estimates for high- and medium- child mortality settings (unpublished data).

### Poliovirus

Two different types of vaccines are available to prevent poliomyelitis: the parenterally administered Salk inactivated polio vaccine, and the live, attenuated oral poliovirus vaccine (OPV; developed via tissue passaging) [19]. While a complete series of either vaccine provides a high level of protection against paralysis, OPV is preferred for routine immunization in high-risk settings and for outbreak response due to its low cost, ease of use, and superior ability to induce intestinal mucosal immunity—required to interrupt transmission. Unfortunately, as the live viruses in first-generation (Sabin-strain) OPV replicate in the gut, their inherent genetic instability can cause them to mutate and recombine with other enteroviruses [46]. In extremely rare cases, vaccine virus that has mutated in the intestines can cause paralysis in a vaccinated child or contact, termed Vaccine-Associated Paralytic Polio (VAPP). Vaccine and vaccine-like viruses that are shed in the feces can also infect unprotected children; in areas of low immunization coverage, long transmission chains can allow mutated viruses to regain neurovirulence. These circulating vaccine-derived polioviruses (cVDPVs) cause outbreaks which must in turn be responded to using OPV, potentially leading to new cVDPVs [46]. In 2021, a new, more genetically stable vaccine was rolled out: novel oral poliovirus vaccine, serotype 2 (nOPV2), constructed with unique attenuating mutations that reduce the likelihood of harmful mutations and recombinations that allow the virus to rapidly regain neurovirulence [47]. Type 2 was prioritized for new vaccine development due to its higher risk of generating cVDPVs, compared with other serotypes; novel OPVs for type 1 and type 3 virus are also under development [48]. Evidence from field use to date has borne out nOPV2’s dramatically lower likelihood of reversion as well as its comparable effectiveness in closing outbreaks, compared with its predecessor Sabin OPV [49].

**Figure 2:**
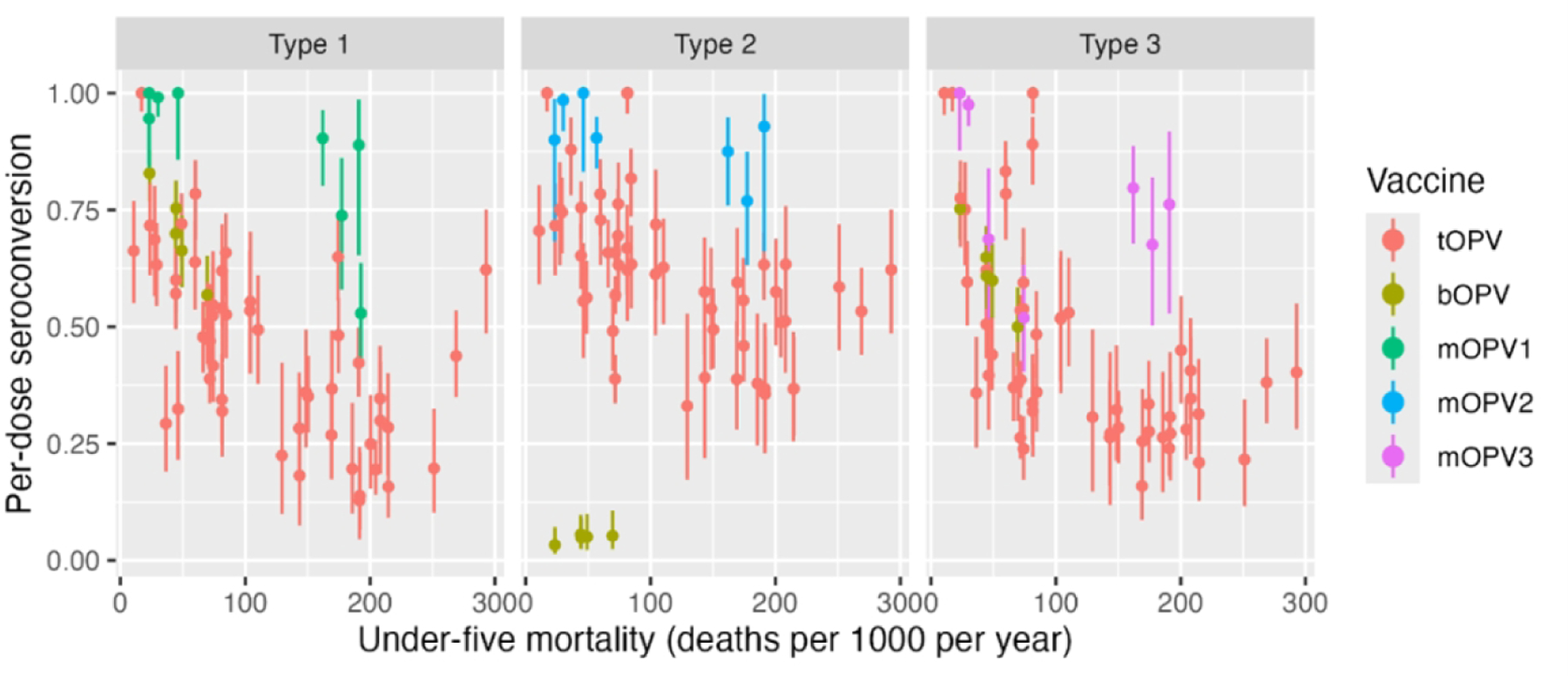
Seroconversion rates in children administered tOPV (from Patriarca et al., [50], Table 1), bOPV (from Macklin et al., [186], Table S1), or mOPV1, 2, 3 (Caceres et al., [53], Table 3) plotted by UNICEF estimated under-five mortality in country and year of study (year of publication used if year of study not reported). Seroconversion adjusted to single dose conversion as 1-(1-S/N)^(1/D), where S is the number seroconverting, N is the number studied, and D is the number of doses administered.

**Figure 3.**
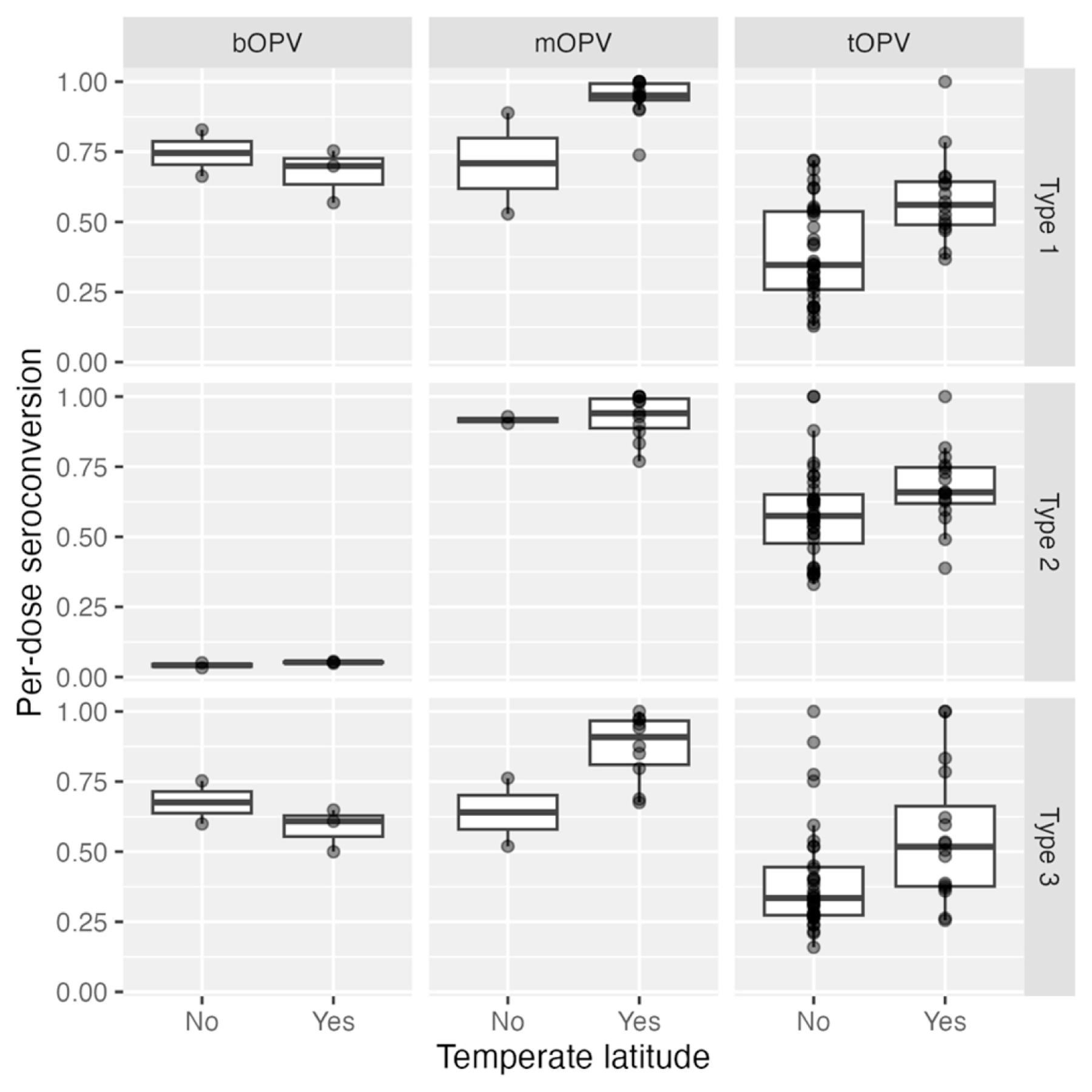
Seroconversion rates in children administered (from Patriarca et al., [50], Table 1), bOPV (from Macklin et al., [186], Table S1), or mOPV1, 2, 3 (Caceres et al., [53], Table 3) plotted by non-temperate (between Tropic of Cancer and Capricorn) or temperate latitude. Seroconversion adjusted to single dose conversion as 1-(1-S/N)^(1/D), where S is the number seroconverting, N is the number studied, and D is the number of doses administered.

Since the 1970s, researchers have observed global variation in OPV performance [50, 51]. Patriarca and colleagues contrasted low prevalence of serum neutralizing antibody activity after 3 doses of trivalent OPV in “developing” countries with higher prevalence in industrialized countries (e.g., US, Canada, Europe), observing a weighted average of 73%, 90%, and 70% prevalence for types 1, 2, 3, respectively. By contrast, a study in the US demonstrated >97% seroprevalence for all three types after three doses of tOPV [52]. (Figure 2) The authors identified interference from Sabin 2 and other enterovirus infections as key drivers of impaired OPV immunogenicity in these settings. Another review of serum neutralizing antibody activity after monovalent OPV administration identified similar geographic differences, with type 1 seroprevalence after one dose ranging from 53% in Uganda to 100% in USSR and the Netherlands, type 2 from 77% in Mexico to 100% in USSR, and type 3 from 52% in Brazil to 100% in the Netherlands [53]. The authors note differences between studies in sites in temperate compared to non-temperate latitudes, with lower median seroconversion rates in non-temperate (81, 89, 72%) versus temperate (95, 98, 94%) settings (Figure 3). A later study in Mozambique (non-temperate) also found relatively lower seroconversion following one dose mOPV2 (61%) [54]. More recent studies of nOPV2 have shown similar differences, with a study in the Gambia (classified as low-income) finding significantly lower two-dose seroconversion in infants and children (67% and 74% respectively) [55], compared with results from earlier studies conducted in high- and middle-income countries (98–100% in Panama, 96% in Dominican Republic, 86–90% in Bangladesh) [56–59].

Although global comparisons are limited because effectiveness studies are only feasible in settings with active poliovirus transmission, some case-control studies have identified sub-national differences in OPV effectiveness against poliomyelitis, with lower effectiveness in places that have historically struggled to control poliovirus transmission (Table 2). Grassly and colleagues demonstrated significantly lower effectiveness of tOPV and mOPV1 against WPV1 poliomyelitis in Uttar Pradesh than in the rest of India (11% per-dose effectiveness of 1 dose tOPV in Uttar Pradesh compared to 23% in the rest of India) [60]. Mangal and colleagues showed lower effectiveness of OPVs against WPV1 and WPV3 in northern states of Nigeria relative to southern states [61].

While serum neutralizing antibody titer of ≥1 in 8 has been established as a correlate of protection for poliomyelitis [62], it is difficult to directly compare immunogenicity and effectiveness across settings because clinical immunogenicity studies tend not to be conducted in the same places as effectiveness studies for reasons of feasibility. While seroprevalence surveys are more feasible to conduct in low-income settings, using these data to assess OPV performance requires accurate ascertainment of vaccination history, which can be difficult.

### Cholera

There are three types of oral cholera vaccines (OCVs) commercially available: inactivated whole cell bacteria alone (WC), inactivated whole cell bacteria with recombinant cholera toxin B (WC + rCTB), and live attenuated (LA, developed via genetic modification of a classical strain). The latter two vaccines, WC +rCTB and LA, are approved for use in some high-income countries (HIC) for travelers 2 years and older since 1991 and 2016, respectively, but are not used in public health programs in endemic countries. The first WHO prequalified (2011) WC vaccine, Shanchol^™^ (Shantha Biotechnic, India), is no longer produced. A WC vaccine with an identical composition, Euvichol-Plus® (EuBiologics, Korea) is registered in several endemic countries and has been WHO prequalified since 2015. This vaccine is accessed through the Gavi-supported cholera vaccine stockpile and delivered through mass vaccination campaigns as a two-dose regimen with a two-week interval among those 1 year or older. Several killed and live attenuated OCVs are in preclinical and clinical development [28].

Due to the different indications for these products, they followed different clinical development pathways, either predominantly in HIC or in low-income countries (LIC) where cholera occurs. Consequently, there is very little published data to allow direct comparison of performance of these vaccine types in both settings.

The safety, immunogenicity and efficacy of the WC vaccines have been almost exclusively evaluated among children and adults in cholera-affected LIC settings. Shanchol^™^ was registered and prequalified based on a randomized controlled trial (RCT) in Kolkata, India demonstrating efficacy of 67% in all ages combined after two years of follow-up [63, 64]. Differences in efficacy were observed between age groups, with the lowest efficacy (49%) shown in children 1 – <5 years old. The trend toward lower efficacy in children <5 years was confirmed in a meta-analysis demonstrating a pooled efficacy of inactivated oral cholera vaccines of 30% (95%CI 15–42%) for children <5 as compared to 64% (58–70%) for those ≥5 years [65]. All subsequent similarly composed WC vaccines have been registered and prequalified based on clinical trials conducted in endemic countries and designed to demonstrate immunologic non-inferiority to Shanchol^™^. One exception is a small phase 1 trial in Korean adults; a seroconversion rate (SCR, >4 fold rise) of 95% was observed for both key antigens, Inaba and Ogawa, following the standard two-dose regimen of Euvichol®, a vaccine with an identical composition [66]. In immunogenicity trials using Shanchol^™^, across several cholera-endemic countries, the SCRs were consistent and lower than in the Korean adults. However, among endemic populations and particularly older adults, there is high seroprevalence at baseline, and individuals with high baseline titers are less likely to have an additional 4-fold rise after vaccination [67]. As children under 5 years tend to have lower baseline seropositivity and titers, their SCRs tend to be higher than adults (though adults have higher final titers). Table 3 summarizes seroconversion rates and geometric mean titers (GMT) observed following two doses of Shanchol^™^ in several countries [65, 68–71]. For WC OCVs, the blunted antibody response and lower efficacy seen among young children compared to adults in the same setting may relate to the reduced ability of the immune system of a young child to generate a strong and enduring response to the polysaccharide primary protective antigen OSP [72].

Vaccines based on the combination of inactivated whole cell bacteria and the non-toxigenic component of the Cholera Toxin dimer, CTB (WC +rCTB) have been evaluated in both HIC and LIC (Table 4). Early prototypes of the vaccine were evaluated in a human challenge among 9 US adults and found to have an efficacy of 64% four weeks after completing a three-dose series [73]. In a large, randomized, placebo-controlled trial in Bangladeshi individuals aged 2 – 45 years, efficacy at 6 months was estimated at 85% (95% CI: 62–94), consistent across all age strata. However, at the one-year interval, the cumulative efficacy had dropped to 62% among all participants and 38% among those aged 2–5 years [74, 75]. In Peru, two studies demonstrated conflicting results (86% short-term efficacy among male military recruits, but 0% efficacy in the first year of surveillance in a large community-based randomized trial) [76, 77]. Notably, in the community-based trial, 61% efficacy (86% against cholera requiring hospitalization) was seen during the second season following a third, booster dose [77]. Two studies of the two-dose regimen, in Mozambique and Zanzibar, estimated effectiveness of 84% and 79% respectively [78, 79].

There have been several commercial versions of the LA OCV, all based on the genetically attenuated CVD 103-HgR strain derived from a pathogenic classical Inaba strain 569B [80–83]. Throughout several different challenge trials in US volunteers, high levels of protection ranging from 90 to 95% at 1 month and 79.5–95.4% at 3 months were consistently demonstrated [80–82]. Experience with CVD 103-HgR based vaccines is more limited in LICs. One retrospective cohort study was conducted following a mass vaccination campaign in response to a cholera introduction on the non-endemic Pohnpei island (Federated States of Micronesia). The campaign targeted all individuals older than 2 years and achieved ~45% vaccination coverage; analysis demonstrated 79% effectiveness among all ages combined [84]. However, a large RCT enrolling participants aged 2 – 41 years in Jakarta, Indonesia with a 4-year follow-up did not demonstrate efficacy against medically attended cholera following a single dose of CVD 103-HgR; neither analysis of shorter post-vaccination intervals nor analysis by age strata yielded significant results [85]. In a nested immunogenicity study, there was a negative relationship between baseline titer and fold antibody rise, and SCR was 42% among those with elevated baseline titers compared to 86% among individuals with low baseline titer [85].

## Evidence for Understanding Disparities in Performance

Evidence suggests that maternal, infant, and environmental factors, in addition to characteristics of the vaccines themselves, all may contribute to the poor performance of oral vaccines in LMICs. The influence of these factors spans a timescale that ranges from before the birth of the infant to well after the vaccines have been administered.

### Maternal Factors and Antibody Interference

The most well-studied maternal factors are transplacental and breast milk antibodies [50, 86]. Serum anti-rotavirus IgG that is passively transferred from mother to infant interferes with seroconversion to several live, oral rotavirus vaccines (ORVs) [87–90], and placentally transferred maternal anti-polio IgG has long been known to interfere with seroconversion after OPV, with the magnitude of the effect apparently serotype-specific [50, 91–94]. Given that OCVs are not given below one year of age, transplacental antibodies are not likely to contribute much to observed differences. Indeed, a recent study aimed at determining the half-life of maternal antibodies to multiple antigens in the diphtheria, pertussis and tetanus vaccine estimated the range to be between 28.7 and 35.1 days, irrespective of demographic or antigen factors [95]. These data provide a rationale for delaying the first dose of vaccination with ORV or OPV as a strategy to potentially reduce the interference from transplacental IgG. However, studies comparing seroconversion rates between children receiving the Rotarix vaccine at 6 and 10 weeks versus 10 and 14 weeks did not consistently show improvements in response [96]; a potential exception is a study in Bangladesh which found higher-than-expected efficacy for Rotarix given on a 10 and 17 week schedule, though there was no early-schedule arm for comparison [97]. Historical analysis did not find significant differences in performance of OPV when the first dose was given at 8 compared to 6 weeks [50], and OPV continues to be given on an early schedule in many countries to achieve early protection for infants and align with the primary series of other antigens. Baseline antibody levels may also be elevated due to natural infection; indeed, some work suggests that failure to fully account for factors affecting susceptibility (including natural infection) could partially explain lower efficacy and effectiveness estimates for ORVs in high-burden settings [98]. Similarly, interference from antibodies resulting from prior exposure to cholera or related antigens is postulated to be a major reason that the LA OCV demonstrated no efficacy in the endemic population of Jakarta, but high efficacy or effectiveness in human challenge studies and in the non-endemic population of Pohnpei, Micronesia [76]. Although it is increasingly recognized that other immune cells (e.g., T lymphocytes, macrophages) can also be transferred across the placenta and may play a role in protecting infants from infection by pathogens [99], their role in infant response to vaccines has not been systematically studied.

Human breast milk is a rich source of bioactive compounds, including secretory IgA as well as other immunologic factors, growth factors, and other non-nutritive elements [100]. Several studies have demonstrated significant differences in the levels of anti-rotavirus IgA and neutralizing antibodies in human milk in samples collected from mothers in LMICs compared to high-income countries [89, 101–107]. Colostrum (compared with other milk stages) has been demonstrated to be highest in anti-polio IgA [50]. Withholding breast feeding has therefore evaluated as an intervention to improve vaccine response. However, there is little evidence for impact for ORVs [108, 109], with one study in Pakistan even showing higher response in infants who were breastfed immediately post vaccination [110]. Furthermore, a recent study evaluating maternal anti-rotavirus antibody levels between India, UK and Malawi showed similar or higher antibody levels in the UK compared to India, yet vaccine virus shedding and seroconversion rates were significantly higher in the UK [111]. Similarly, although some early research suggested an inhibitory effect of frequent breastfeeding on OPV response, particularly in the first days of life [50, 112], later research has found very little effect [50, 113, 114] or even a positive association [115, 116]. Given the older age at which cholera vaccines are typically given, few studies have assessed the impact of withholding breastfeeding. However, one study in Bangladesh found that vibriocidal responses were higher among older infants given buffered Dukoral (WC+rCTB) when breastfeeding was withheld for 3 hours prior to vaccination; no differences were seen among younger infants or in IgA or IgG antitoxin responses [117]. Bioactive non-antibody components in human milk such as lactoferrin, lactadherin, and tenascin-C have also been considered for their effects on ORV response. However, the few systematic assessments of these factors in LMIC populations have demonstrated conflicting outcomes [105, 118, 119]. *In vitro* studies also indicate the potential for human milk oligosaccharides (HMO) to influence rotavirus vaccine replication, and this remains to be validated in population studies [120]. HMOs and other breast milk components have not been studied for their influence on OPV or OCV response. However, one study found that infants receiving formula supplemented with bovine milk oligosaccharides had a higher fecal IgA response to OPV compared with infants receiving conventional formula; the authors speculated that this effect was mediated through differences in the infant gut microbiome [121].

### Infant and maternal-infant factors

Genetic differences in the expression of histo-blood group antigens (HBGAs) may play a role in differences in ORV response. Specifically, differences in the expression of functional fucosyltransferase (FUT) genes have been evaluated. FUT2 and FUT3 determine the expression of α(1,2) and α(1,3) fucosylated HBGAs that establish individual secretor and Lewis status, respectively. These glycans serve as cellular attachment factors for several human rotaviruses in a VP4 genotype-dependent manner and thus can influence susceptibility to live ORVs as well as wild-type rotavirus strains. Several studies have reported lower seroconversion rates to Rotarix vaccine in non-secretor infants (lacking a functional FUT2 gene), while the effects on vaccine shedding have been less clear [122–129]. The association of the Lewis A phenotype with reduced seroconversion or vaccine shedding has also been described in some studies. There are fewer studies on the effect of secretor status and Lewis status on other licensed rotavirus vaccines [130–132]. Outside the context of VP4-glycan interactions, differences in HBGA expression are also associated with differences in microbiome and HMO composition, and thus may indirectly influence vaccine response. Two studies have determined the effect of maternal secretor status on infant rotavirus vaccine response but with conflicting results, possibly related to the underlying differences in the study populations given that one study was conducted in the United States and the other in Bangladesh [123, 133]. In contrast, HBGAs have not been found to play a role in OPV immunogenicity [134]. Secretor status has not been found to significantly affect OCV immunogenicity [135]. Although O blood type has been associated with increased risk for severe cholera disease, the evidence for the effect of O blood type on immune response has been mixed [136–138], perhaps related in part due to differences in the vaccines studied and the mechanism by which they stimulate an immune response. Small studies have found genes potentially associated with development of paralytic polio [139] or susceptibility to cholera [140], but these have yet to be studied in relation to vaccine response.

Nutritional status is another infant factor that may affect vaccine response. A review of rotavirus vaccine performance found that vaccine effectiveness and efficacy point estimates were 37–64% lower in malnourished versus well-nourished children, when categorized by length-for-age (a measure of chronic undernutrition) [141]. Several studies have also noted reduced immunogenicity of OPV in children with chronic malnutrition [116, 142, 143]. Few similar studies have been conducted for cholera vaccines, but no association between chronic undernutrition and response to Dukoral or CTB subunit was found in two small studies in Bangladesh [144, 145]. Micronutrients have also been investigated for their effect on oral vaccine response. Despite the established effects of vitamin A on overall child health, and linkages of vitamin D and immune function, evidence to-date is limited and has not supported a clear relationship between these micronutrients and response to ORV, OPV, or OCV [146–148]. Although zinc has not been found to be associated with response to ORV [148, 149] or OPV [150], some studies have suggested a positive effect of zinc on vibriocidal immune response to killed cholera vaccine [117, 151, 152]. Nonetheless, meta-analysis has not found a significant effect of micronutrient supplementation on oral vaccine response [146]. Nutritional status is also highly intertwined with other social and environmental factors, including co-infections and their impact on gut health, making this a complex area of study.

### Environmental factors

For live vaccines that replicate in the infant gut, the intestinal microenvironment is likely to play a critical role in vaccine response. Significant associations have been described between markers of environmental enteric dysfunction (EED) and lack of response to ORVs [115, 153, 154] or impaired response to OPV [115, 155] or live-attenuated (LA) OCV in some studies [156]. EED is thought to be the result of repeated enteric infections in settings of low hygiene and sanitation infrastructure and is characterized by increased inflammation as well as changes in intestinal architecture including villous blunting and crypt hyperplasia. Although a number of biomarkers have been studied, none are widely accepted as diagnostic, except intestinal biopsy. While it is logical to infer that EED would be unfavorable for the replication of live vaccines, increased seroconversion to ORVs in children with markers associated with EED has also been described [157, 158]. A positive association between markers of EED response to WC OCV has also been reported in children, suggesting a complex relationship among EED, inflammation, and immune response to mucosal vaccines [144]. In terms of microbial ecology at the time of vaccination, the co-administration of other vaccines (OPV influence on ORV being the most-studied example), differences in the intestinal microbiome and virome, as well as concurrent infections have all been evaluated as factors influencing oral vaccine response in different studies. While the extent to which OPV interferes with ORV response has varied among studies, a large analysis pooling data from 33 high- and low-childhood mortality regions showed that OPV co-administration is significantly associated with reduced seroconversion to ORVs [159]. In contrast, OPV immunogenicity does not appear to be affected by ORV co-administration [146, 160–168].

In one study of co-administration of WC OCV and OPV in toddlers (aged 1 – 3 years), immune responses to both vaccines were comparable in the co-administration and single administration groups [169]. The presence of non-polio enteroviruses has also been associated with poor response to ORVs [170, 171] as well as poor responses to OPV in multiple countries [170, 172, 173], but has not been well studied for OCVs. There are also conflicting results from different studies on the association of intestinal microbiome composition and oral vaccine response. Correlations between presence of *Enterobacteriaceae* and seroconversion to ORVs have been described in Ghana and Pakistan [171, 174]. However, these differences were not seen in a cohort from India, UK, and Malawi [111, 175]. In studies conducted in Bangladesh and China, increased prevalence of *Actinobacteria* (such as *Bifidobacteria)* was positively associated with OPV response, but no impact was found in a cohort of infants in South India [173]. The relationship of other co-infections or microbiome diversity to OPV response appears complex, with interpretations complicated by small sample sizes [170, 176–178]. Few comparable data exist for OCVs, and although helminth burden has been negatively associated with response to cholera infection, antihelminthic treatment has not been shown to improve OCV response [146, 179, 180]. Finally, other environmental factors that impact oral vaccine response include differences in access to water, sanitation, and hygiene that may result in a higher force of infection and higher rates of other enteric infections in LMICs.

### Vaccine Factors

Vaccine-related factors, including dose and strain, may also play a role in overall vaccine performance. Increasing the dose titer has been explored as a way to improve vaccine immunogenicity, but without supportive evidence to date for ORV [181], and with a small effect demonstrated via meta-analysis for OPV and OCV [146]. Administration of a booster dose of ORV has also been associated with increased immune response [182], while for OPV, a birth dose is recommended to secure immune response in high-risk populations [183]. Limited data are available to determine the value of additional doses of OCV. Strain or genotype mismatch has also been suggested as a possible mechanism for differing vaccine performance. Analysis has shown that ORVs are slightly less effective against heterotypic versus vaccine strains of rotavirus [184, 185]. OPV and OCV protect against circulating serotypes and serogroups, and the effects of genetic variations on vaccine performance have not been comprehensively studied.

## Conclusions

Despite the tremendous successes of oral vaccines against rotavirus, polio, and cholera, their performance is still limited in LMICs among young children. Although performance disparities have been well described, they are not as well understood. Overall, these data highlight the rather ambiguous and often contradictory nature of evidence on factors affecting oral vaccine response, cautioning against broad extrapolation of outcomes based on one population or one vaccine type. Indeed, a systematic analysis of interventions aimed at improving oral vaccine response found little evidence to support withholding breastfeeding, vaccine buffering, or narrowing the dose window between vaccines [146]. Even interventions well supported by evidence, such as separating oral rotavirus and oral poliovirus vaccine administration, may not be feasible programmatically. In addition, meaningful impact on performance of oral vaccines will likely only be possible with a suite of interventions, given the complex and multifactorial nature of the problem and our lack of complete understanding. Development and deployment of new vaccines and innovative precision population health approaches that incorporate region-specific environment and host factors may be required to successfully improve the performance of oral vaccines in LMICs.

## Supplementary Material

1

## Figures and Tables

**Table 1:** Licensed oral rotavirus vaccines and their characteristics, 2024

Vaccine	Manufacturer	Genotype(s)	Formulation	WHO-prequalification (year)	Number of doses	Schedule
RotaTeq	Merck	G1, G2, G3, G4, P[8]	Liquid	Yes (2008)	3	Dose 1 starting at 6 to 12 weeks of age; Subsequent doses at 4- to 10-week intervals; Dose 3 should not be given after 32 weeks of age
Rotarix	GSK	G1P[8]	Liquid	Yes (2009)	2	Dose 1 starting 6 weeks; Dose 2 at least 4 weeks after dose 1 and up to 24 weeks of age
Rotavac	Bharat Biotech International Limited	G9P[11]	Liquid (frozen and non-frozen)	Yes (2018)	3	Dose 1 starting at 6 weeks of age; Subsequent doses at 4 week intervals; Dose 3 should not be given after 34 weeks of age
Rotasiil	Serum Institute of India	G1, G2, G3, G4, G9	Liquid, Lyophilized	Yes (2018)	3	Dose 1 starting at 6 weeks of age; Subsequent doses at 4- week intervals; Dose 3 should not be given after 34 weeks of age
Rotavin-M1	Polyvac	G1P[8]	Liquid (frozen and non-frozen)	No (NA)	2	Dose 1 starting at 6 weeks of age; second dose after 60 days and up to 6 months of age
Lanzhou Lamb Rotavirus	Lanzhou Institute of Biological Products	G10P[15]	Liquid	No (NA)	3	Dose 1 starting at 2 months of age; Annual dose up to 3 years of age
Lanzhou Lamb Rotavirus3	Lanzhou Institute of Biological Products	G2, G3, G4	Liquid	No (NA)	3	Dose 1 starting at 2 months of age; Annual dose up to 3 years of age

**Table 2.** Matched case-control studies of oral polio vaccine (OPV) effectiveness against wild poliovirus type 1 (WPV1) poliomyelitis

Paper	Years	Location	Vaccine	Matched cases	Effectiveness^d^
Balraj 1990^e^	1988	Tamil Nadu, India	OPV (3 doses relative to 0, type not specified)	69	62 (27–80)^c^
Sutter 1991^e^	1988–1989	Oman	OPV (3 doses relative to 0, type not specified)	70	91 (36–99)
Deming 1992^e^	1986	The Gambia	tOPV (3 or more doses, relative to 0–2)	164	72 (57–82)
Grassly 2007	1997–2006	Uttar Pradesh, India	tOPV (1 dose, relative to 0)	1499	11 (7–14)^a^
		Bihar, India		204	19 (8–29)^a^
		Rest of India		399	23 (17–29)^a^
Jenkins 2008	2001–2007	North-west Nigeria	tOPV (1 dose, relative to 0)	925	13 (6–19)^b^
		North-east Nigeria		158	23 (7–37)^b^
		North-central		68	23 (1–40)^b^
		Nigeria			
		Southern Nigeria		23	54 (4–78)^b^
O’Reilly 2012	2001–2011	Pakistan & Afghanistan	tOPV (1 dose, relative to 0)	883	13 (6–19)
Mangal 2014	2001–2012	Northern Nigeria	tOPV (1 dose, relative to 0)	1307	19 (16–23)^b^
		Southern Nigeria		63	36 (21–57)^b^
Mahamud 2014	2013	Somalia	OPV (1–3 doses relative to 0, type not specified)	99	59 (2–83)
Chard 2021	2010–2020	Afghanistan	OPV (1 dose, any type)	249	19 (15–22)

a Assuming 100% routine immunization coverage

b Assuming 0% routine immunization coverage

c Unmatched case-control analysis

d Vaccine effectiveness against WPV1 poliomyelitis

e Controls not test-negative acute flaccid paralysis cases.

**Table 3:** Comparison of Seroconversion Rate and Geometric Mean Titer (GMT) following two doses of Shanchol among adults and children under 5 years of age in cholera endemic countries

		Adults (18 years and above)						Children (1–5 years)					
		O1 Inaba			O1 Ogawa			O1 Inaba			O1 Ogawa		
		N	Seroconversion rate [95% CI]	GMT [95% CI]	N	Seroconversion rate [95% CI]	GMT [95% CI]	N	Seroconversion rate [95% CI]	GMT [95% CI]	N	Seroconversion rate [95% CI]	GMT [95% CI]
**India^a^**	Baseline	37	-	186 [93, 370]	34	-	377 [211, 673]	12	-	24 [6, 100]	7	-	20 [1, 377]
	Post second dose		46% [31, 62]	1003 [763, 1319]		41% [26, 58]	1204 [858, 1690]		92% [65, 99]	604 [311, 1172]		86% [49, 97]	525 [116, 2384]

**Bangladesh^b^**	Baseline	416	-	44 [38, 51]	416	-	60 [52, 69]	214	-	19 [15, 23]	214	-	17 [14, 21]
	Post second dose		83% [79, 86]	435 [390, 485]		73% [69, 77]	375 [354, 440]		86% [81, 90]	354 [288, 431]		85% [79, 89]	263 [215, 323]

**Philippines^c^**	Baseline	376	-	36 [35,37]	376	-	74 [73, 75]	74	-	4 [4, 5]	74	-	5 [4, 5]
	Post second dose		76% [72, 81]	732 [727,737]		74% [69, 78]	962 [957, 967]		84% [73, 91]	159 [147,171]		86% [77, 93]	283 [271, 295]

**Nepal^d^**	Baseline	312	-	24 [18, 32]	312	-	52 [40, 70]	238	-	4 [3, 5]	238	-	4 [3, 6]
	Post second dose		79% [75, 83]	620 [523, 735]		75% [71, 78]	1036 [896, 1197]		94% [92, 97]	398 [319, 496]		94% [91, 97]	604 [486, 725]

**Ethiopia^e^**	Baseline	53	-	17 [9, 32]	37	-	24 [10, 55]	17	-	3 [1, 11]	16	-	3 [1, 9]
	Post second dose		81% [69, 89]	254 [160, 403]		70% [54, 83]	306 [160, 587]		53% [31, 74]	15 [4, 51]		75% [51, 90]	73 [25, 217]

Abbreviations:CI=confidence interval; GMT=geometric mean titers; N=number of participants analyzed.

The seroconversion rate is the proportion of participants with at least 4-fold rise vibriocidal titers against Vibrio cholerae O1 Inaba and O1 Ogawa from baseline to post second dose. Vibriocidal assay performed in different laboratories for each trial using similar methods.

a Shanchol trial, post dose 2 Shanchol only

b Cholvax trial, post dose 2 Shanchol only

c Euvichol trial, post dose 2 Shanchol only

d Euvichol-S trial, post dose 2 Shanchol only

e Shanchol trial, post dose 2 Shanchol only

**Table 4:** Comparison of Efficacy and Effectiveness Estimates of Inactivated Whole Cell Bacteria with Recombinant Cholera Toxin B (WC+rCTB) and Live-attenuated (LA) Vaccines in High-Income Countries (HIC) and Low-Income Countries (LIC)

**WC +rCTB vaccines**						
	**HIC**			**LIC**		
	**USA (challenge study) 1 Month**		**Matlab, Bangladesh 12 Months**	**Peru, Military 5 Months**		**Pampas, Peru 12 Months**
**Efficacy**	64%^*^		62%^†^	86%		0%^‡^
			**Biera, Mozambique 5 Months**		**Zanzibar, Tanzania 15 Months**	
**Effectiveness**			84%^§^		79%^∥^	

**Live Attenuated Vaccines based on CVD 103**						
	**HIC**		**LIC**			
	**USA (challenge study) 1 Month**		**Jakarta, Indonesia**			
	Orochol (2 – 10 × 10^8^𝐶𝐹𝑈)	Vaxchora (4 × 108 𝑡𝑜 2 × 10^9^𝐶𝐹𝑈)	Orochol-E (~5 × 10^9^𝐶𝐹𝑈)			
**Efficacy**	79.0%^¶^	79.5%^#^	14%^**^			
			**Pohnpei, Micronesia**			
			Orochol-E (~5 × 10^9^𝐶𝐹𝑈)			
**Effectiveness**			79.2%^††^			

* 4 weeks post three dose vaccination

† 1 year post vaccination with three doses

‡ 1 year post vaccination with two doses

§ 6 months post one or two dose vaccination

∥ 1 year post vaccination with two doses

¶ based on prevention of >3L diarrhea three months post vaccination

# based on prevention of >3L diarrhea three months post vaccination

** no statistically significant evidence of efficacy throughout 4 years of follow-up

†† 3-month post vaccination

## Data Availability

All data reported are publicly available.
