# Supplementary material for: Geographic disparities impacting oral vaccine performance: Observations and future directions
Source: Clin Exp Immunol. Author manuscript; Available in PMC 2025 Oct 13. (PMC11773816; doi:10.1093/cei/uxae124)

SUPPLEMENTAL MATERIAL

Figure S1. Seroconversion rates in children administered tOPV (from Patriarca et al. 1991 Table 1), bOPV (from Macklin et al. 2019 Table S1), or mOPV1, 2, 3 (Caceres et al. 2001 Table 3) plotted by World Bank income group of study country (2023 classification). Seroconversion adjusted to single dose conversion as 1-(1-S/N)^(1/D), where S is the number seroconverting, N is the number studied, and D is the number of doses administered. LIC = low-income country, LMC = lower-middle-income country, UMC = upper-middle-income country, HIC = high-income country.

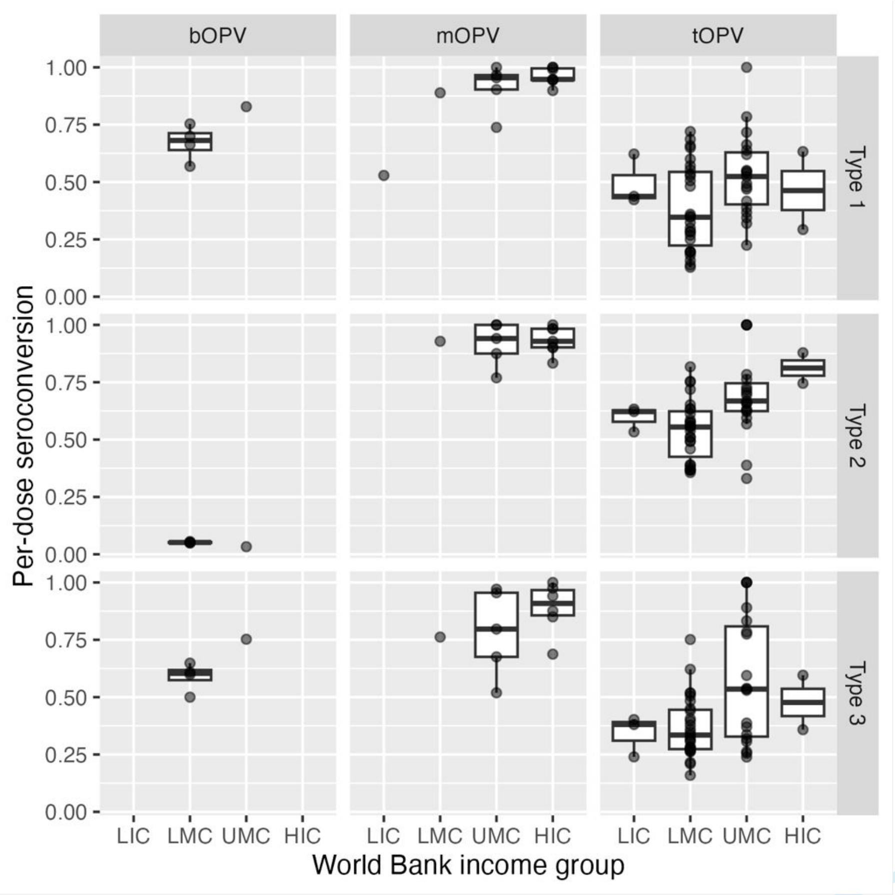

Supplement: 1 [file NIHMS2107391-supplement-1.pdf]
